# Construction and Growth Differences in Mother Bamboo Ramet Systems of Typical Monopodial Bamboos Under Different Planting Densities

**DOI:** 10.3390/plants15142169

**Published:** 2026-07-15

**Authors:** Guibin Gao, Xing Wen, Fangyuan Bian, Zhizhuang Wu, Jinfang Qian, Yiji Huang, Hao Zhong, Yanhong Pan, Xiaoping Zhang

**Affiliations:** 1 China National Bamboo Research Center, Hangzhou 310012, China; anshu998@caf.ac.cn (G.G.); wenxing202202@163.com (X.W.); bianfangyuan@caf.ac.cn (F.B.); wzzcaf@126.com (Z.W.); zhonghao0726@163.com (H.Z.); zhukan2004@163.com (Y.P.); 2 Long-Term Observation and Research Station for Farmland Shelterbelt Ecosystem in Hangzhou-Jiaxing-Huzhou Plain, Hangzhou 310012, China; 3 Changxing County Natural Resources and Planning Bureau, Huzhou 313100, China; 15869175672@163.com; 4 Shuikou Township Forestry Technology Extension Station, Changxing County, Huzhou 313108, China; zxpyg2016@126.com

**Keywords:** ramet system, *Phyllostachys edulis*, *Phyllostachys praecox*, underground rhizome, bud bank, branching

## Abstract

Bamboo forests are formed by the interlacing of multiple ramet systems. However, the interaction relationships between ramet systems remain unclear. To determine the effects of initial planting density on the construction of ramet systems in typical monopodial bamboos, and to clarify the differentiation rules of underground clonal architecture for bamboo species with different culm diameters, in this study, we selected large-diameter species *Phyllostachys edulis* and small-diameter species *Phyllostachys praecox* as study species. A pot experiment with root restriction was conducted using three density gradients of low, medium, and high. Rhizome morphology, underground bud bank dynamics, branching types, and the spatial distribution of the ramet systems were determined. The results showed that density significantly affected rhizome elongation, node allocation, the ratio of dormant buds to germinated buds, and branching hierarchy distribution of ramet systems, with pronounced differences between species. In *P. edulis*, longer rhizomes, higher dormant bud accumulation, and the branching hierarchies concentrated in low-to-moderate grades were observed at low density. In contrast, rhizome growth and branching were significantly inhibited with increasing density. For *P. precox*, multistage rhizome extension, higher sprouting activity, and wider branching distribution in the middle and posterior segments of the rhizomes occurred under low- and medium-density conditions. Under high density, however, the peak of dormant buds shifted backward, and a sprouting compensation effect occurred. The initial density reshaped the spatial architecture of ramet systems by altering the intensity of underground competition and preferentially inhibiting the development of new branches and high-grade rhizomes. This study focused on monopodial bamboos during the early establishment stage of mother bamboo development. First, it explored density-driven effects governing ramet system formation. Second, it elucidated contrasting clonal trade-off responses between large- and small-diameter bamboo species. The results will supplement theories on the population construction of woody clonal plants. In addition, they can guide rational close planting and targeted regulation of ramet systems in artificial bamboo stands.

## 1. Introduction

Clonal plants achieve ramet proliferation and spatial colonization via organs, such as underground rhizomes and stolons [1,2]. The mechanisms underlying the construction, expansion, and maintenance of ramet systems have long been core scientific issues and a key research focus in clonal and plant population ecology. Since the proposal of the genet-ramet structure theory [3], it has been widely recognized that clonal plants are not simple assemblages of independent individuals but are composed of clonal networks with genetically identical, physiologically integrated, and functionally specialized ramets [4,5]. Their survival and expansion are highly dependent on resource sharing, risk spreading, and functional trade-offs among ramets [6,7]. Population density, a key factor driving the differentiation of resource allocation and growth strategies in clonal plants, induces species to adopt ecologically favorable trade-offs by regulating intraspecific competition intensity, resource acquisition efficiency, and microhabitat heterogeneity [8,9]. This tradeoff represents an evolutionarily stable strategy that balances clonal growth efficiency, competitive ability, and dispersal risk.

To date, most prior studies on density and clonal responses have focused on herbaceous clonal plants [10,11,12], clarifying the regulatory effects of density on ramet quantity, clonal architecture, biomass allocation, bud bank patterns, resource capture efficiency, and physiological integration intensity. However, these conclusions are highly dependent on herbaceous systems. This makes it difficult to extrapolate them to woody rhizomatous clonal plants. Previous studies on density gradient clonal growth responses in woody bamboo species have preliminarily confirmed that density can significantly alter bamboo growth and asexual expansion characteristics [13,14]. Nevertheless, most studies on density responses in bamboo forests have taken entire populations as research objects. Few investigations focus on how density affects the growth of bamboo ramet systems. Research on woody clonal plants remains in its infancy, with far less depth and systematicity than research on herbaceous taxa.

Monopodial bamboos, for example, *Phyllostachys edulis* and *Phyllostachys praecox*, are typical woody rhizomatous clonal plants [15,16]. Population construction relies on a complete clonal process: mother bamboo establishment, underground rhizome expansion, ramet new bamboo sprouting, and the turnover of old and new individuals [17]. A single-mother bamboo undergoes horizontal extension via underground rhizomes, growing several meters annually. Buds on rhizome nodes differentiate into new rhizomes or shoots, which develop into new bamboo, forming complex clonal networks characterized by interconnected rhizomes and bamboo, nested age classes, and strong spatial heterogeneity. In this system, population density regulates aboveground ramet competition and profoundly affects the branching pattern, extension direction, biomass allocation, and nutrient transport efficiency of underground rhizomes [18], determining the spatial distribution, age structure, and productivity of the entire bamboo forest.

As woody clonal plants in the subtropical regions of China, monopodial bamboo forests are composed of groups of interacting ramet systems with genetically homologous and independently connected rhizomes, rather than individual assemblages [19]. Field experiments and investigations have confirmed that competition, isolation, and complementarity effects among ramet system groups profoundly influence bamboo forest productivity, underground rhizome network distribution, and population stability [20]. However, current research lags considerably regarding the dynamic processes of ramet systems, from mother bamboo establishment to rhizome expansion, and their density regulatory effects in monopodial bamboos.

This hinders our understanding of the formation processes of woody clonal population structures and the maintenance mechanisms of bamboo forest ecosystem stability. It also restricts theoretical support and technological innovation for sustainable bamboo forest management, for example, density regulation, structural optimization, and degraded bamboo forest restoration. The underground rhizome system is the core functional unit for ramet expansion in monopodial bamboos [21,22]. Its morphological architecture, bud bank dynamics, and branching pattern determine the growth potential and spatial occupancy capacity of ramet systems. Rhizome growth is strictly regulated by species-specific genetic traits and exhibits strong plastic responses to environmental factors such as habitat resource availability [23,24]. Current research lacks an integrated analysis of rhizome morphology, bud banks, and branching patterns of ramet systems, especially comparative studies on typical large-diameter and small-diameter monopodial bamboos under density gradients. This makes it difficult to clarify the formation processes of developmental differences in the ramet systems of bamboo species with different clonal strategies.

*Phyllostachys edulis* and *Phyllostachys praecox* are typical representatives of large- and small-diameter monopodial bamboos in subtropical China and are ideal materials for exploring the developmental rules and density response effects of ramet systems in monopodial bamboos [19,25]. A pot experiment with root restriction was conducted using low-, medium-, and high-planting-density gradients. The rhizome morphology, underground rhizome bud bank, branching type, and spatial distribution of the mother bamboo ramet systems of the two monopodial bamboos at different densities were determined. We aimed to explore how initial density shapes monopodial bamboo ramet systems and then unpack the internal effects that create different clonal strategies among typical monopodial bamboos. This work expands existing theories on ramet system development in woody clonal plants. Our results also supply practical guidance for optimized close planting, precise ramet control, and sustainable management of artificial bamboo forests.

## 2. Results

### 2.1. Rhizome Morphology of Mother Bamboo Ramet Systems

**Figure 1:**
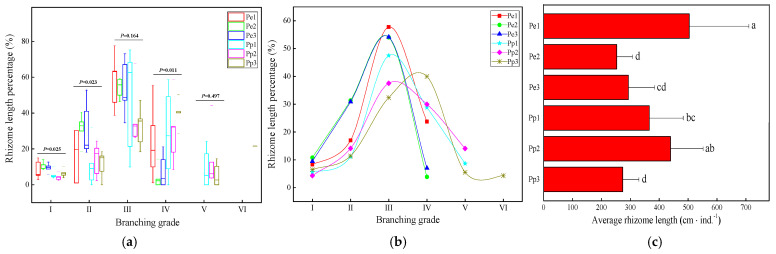
Rhizome length of ramet systems. (**a**) Percentage of rhizome length at different branching grades; (**b**) Variation trend of rhizome length percentage across branching grades; (**c**) Average rhizome length of different ramet systems. Rhizomes were classified by branching order. Different letters indicate significant differences (*p* < 0.05). The initial branch of the ramet system was designated as Grade I, branches sprouting from Grade I branches as Grade II. Three density gradients were set for *P. edulis*: low density (Pe1), medium density (Pe2), and high density (Pe3), with one, two, and three mother bamboos planted per pot, respectively. *P. praecox*: low density (Pp1), medium density (Pp2), and high density (Pp3), with the same density settings as *P. edulis*.

Rhizome length proportions across branching grades differed significantly between *P. edulis* (Pe) and *P. praecox* (Pp) and among density treatments (Grade I, II, IV: *p* < 0.05; Grade III peak showed no interspecific difference, *p* > 0.05; Figure 1a). Pe allocated more rhizome length to low–moderate grades, while Pp produced more high-grade branches. Rhizome length percentage followed a consistent unimodal curve peaking mainly at Grade III for both species (Figure 1b). Low-density Pe1 exhibited the highest Grade III peak with suppressed high-grade rhizome growth; Pp’s peak value was lower and declined gently with sustained branching across grades. Rising planting density reduced the peak rhizome length proportion for both taxa via intensified intraspecific competition. Species and density jointly regulated average rhizome length with divergent responses (Figure 1c). Pe rhizome length declined markedly with increasing density (*p* < 0.05), with low density being optimal for underground elongation. By contrast, Pp tolerated moderate competition and maximized rhizome growth under medium density; only high density significantly restricted its extension (*p* < 0.05).

**Figure 2:**
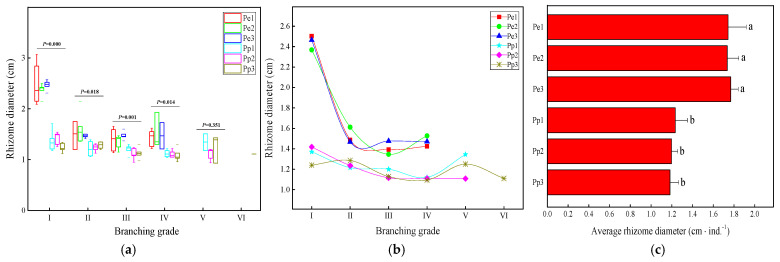
Rhizome thickness of ramet systems. (**a**) Rhizome thickness at different branching grades; (**b**) Variation trend of rhizome thickness across branching grades; (**c**) Average rhizome thickness of different ramet systems. Different letters indicate significant differences (*p* < 0.05).

Rhizome diameter consistently differed between species across all branching grades I–IV, with Pe bearing markedly thicker rhizomes than Pp (*p* < 0.05; Figure 2a). Pe rhizome diameter decreased sharply with rising branch grade and stabilized after Grade III, whereas Pp maintained uniformly thin rhizomes with minor fluctuations throughout all grades (Figure 2b). Density exerted no significant effect on average rhizome diameter for either species (*p* > 0.05; Figure 2c).

**Figure 3:**
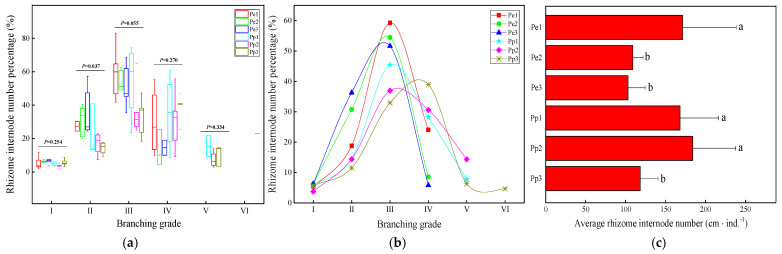
Rhizome nodes of ramet systems. (**a**) Percentage of rhizome nodes at different branching grades; (**b**) Variation trend of rhizome node percentage across branching grades; (**c**) Average rhizome nodes of different ramet systems. Different letters indicate significant differences (*p* < 0.05).

Rhizome node percentages concentrated in Grades II–IV branches for both bamboo species (Figure 3a). Under low and medium densities, Pe dominated Grades II–III node allocation; Pp extended nodes to Grades V–VI and displayed stronger multistage branching. All Pe treatments presented a unimodal node percentage curve peaking at Grade III, and higher density compressed node distribution toward low–moderate grades (Figure 3b). For Pp, the node peak shifted backward to Grade IV with rising density, maintaining abundant high-grade nodes and reflecting divergent clonal allocation strategies. Total rhizome node counts responded differently to density (Figure 3c). Pe1 held the maximum total nodes, which dropped significantly as density increased (*p* < 0.05), indicating high density sensitivity. Pp retained abundant nodes under low and medium densities, with significant inhibition only at high density.

### 2.2. Underground Rhizome Bud Bank of Mother Bamboo Ramet Systems

**Figure 4:**
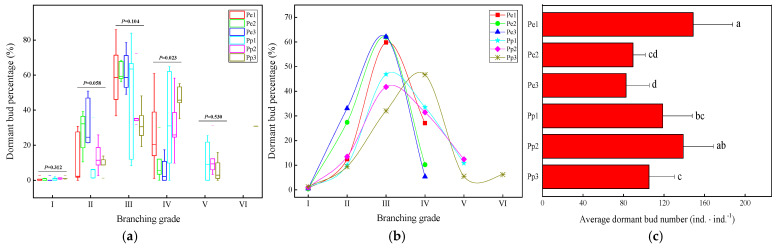
Dormant buds of ramet systems. (**a**) Percentage of dormant buds at different branching grades; (**b**) Variation trend of dormant bud percentage across branching grades; (**c**) Average number of dormant buds in different ramet systems. Different letters indicate significant differences (*p* < 0.05).

Dormant bud proportions remained negligible in Grade I, peaked at Grade III, and declined sharply from Grade IV onward (Figure 4a). All Pe treatments formed a Grade III dormant bud peak; Pp’s peak shifted to Grade IV under high density (Pp3), indicating delayed bud dormancy and a prolonged developmental window (Figure 4b). Pe accumulated far more dormant buds overall than Pp. The total dormant bud number of Pe decreased significantly with density growth (*p* < 0.05), peaking at Pe1; Pp’s dormant bud quantity was maximized under medium-density Pp2 (Figure 4c).

**Figure 5:**
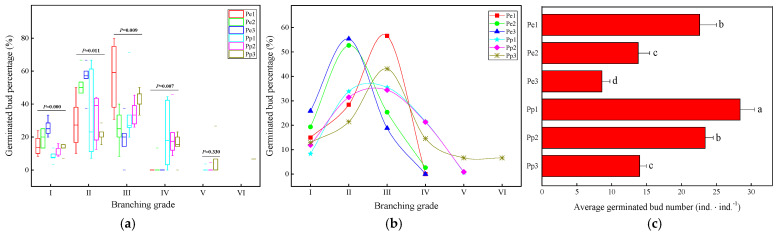
Germinating buds of ramet systems. (**a**) Percentage of germinating buds at different branching grades; (**b**) Variation trend of germinating bud percentage across branching grades; (**c**) Average number of germinating buds in different ramet systems. Different letters indicate significant differences (*p* < 0.05).

Germinated bud percentages rose to Grades II–III maxima and fell to near-zero values in Grades V–VI (Figure 5a). Pe1 showed superior early sprouting, with germination rapidly suppressed in high grades; Pp sustained high germination across mid-to-late branches. Both species followed unimodal germination curves (Figure 5b). Higher density advanced Pe’s germination peak from Grade III (Pe1) to Grade II (Pe2, Pe3). At high density (Pp3), Pp exhibited a germination compensation effect, with the highest peak and longer sprouting duration relative to Pe. Total germinated bud counts declined with rising density for both species, and Pp produced more germinated buds than Pe at identical density levels (Figure 5c).

**Figure 6:**
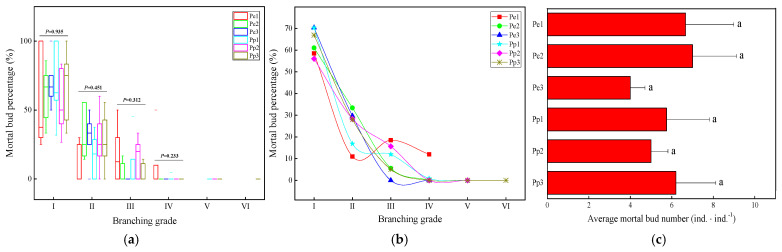
Mortal buds of ramet systems. (**a**) Percentage of mortal buds at different branching grades; (**b**) Variation trend of mortal bud percentage across branching grades; (**c**) Average number of mortal buds in different ramet systems. Same letters indicate no significant differences (*p* > 0.05).

Mortal bud proportions decreased continuously as branching grade increased (Figure 6a), with the highest proportion observed in Grade I and negligible values observed above Grade IV (Figure 6b). Neither bamboo species nor planting density altered mortal bud percentages at single grades (*p* > 0.05; Figure 6a) or the average total mortal bud number (*p* > 0.05; Figure 6c).

**Figure 7:**
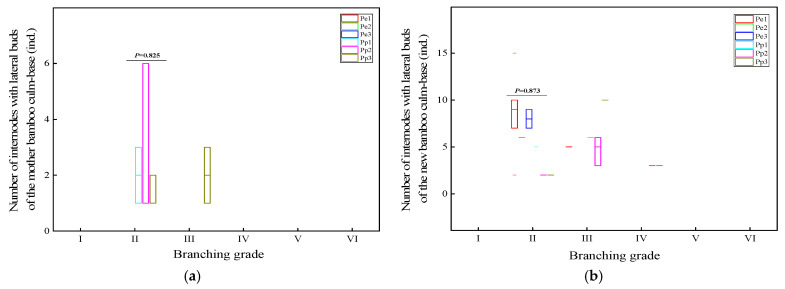
Basal lateral buds of ramet systems. (**a**) Number of mother bamboo basal lateral buds; (**b**) Number of new bamboo basal lateral buds.

Mother bamboo basal lateral buds only appeared in Pp treatments, while new bamboo basal lateral buds existed in both species (Figure 7a,b). Pe allocated more lateral buds to Grade II, whereas Pp concentrated lateral buds in Grade III and generated minor numbers in Grade IV and above. No significant treatment differences occurred within the dominant Grade II distribution zone (*p* > 0.05). Pp activated lateral buds on both mother and new bamboos, while Pe only activated new bamboo lateral buds, revealing inherent interspecific clonal strategy differentiation.

### 2.3. Branching of Mother Bamboo Ramet Systems

**Figure 8:**
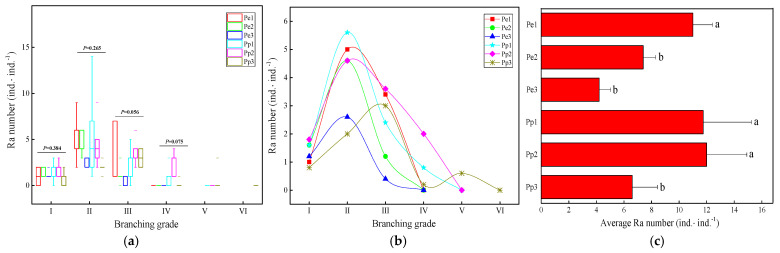
Rhizome branches (Ra) of ramet systems. (**a**) Number of Ra branches at different branching grades; (**b**) Variation trend of Ra branch number across branching grades; (**c**) Average number of Ra branches in different ramet systems. Different letters indicate significant differences (*p* < 0.05).

Rhizome branch (Ra) counts peaked at Grade II and were mainly limited to Grades I–III for both species (Figure 8a). Pp1 and Pe1 possessed the highest Ra peaks, while Pe3 exhibited the lowest (Figure 8b). Average Ra quantities of Pp1, Pp2, and Pe1 were significantly higher than those of Pe2, Pe3, and Pp3 (*p* < 0.05), confirming strong high-density inhibition of total rhizome branching (Figure 8c).

**Figure 9:**
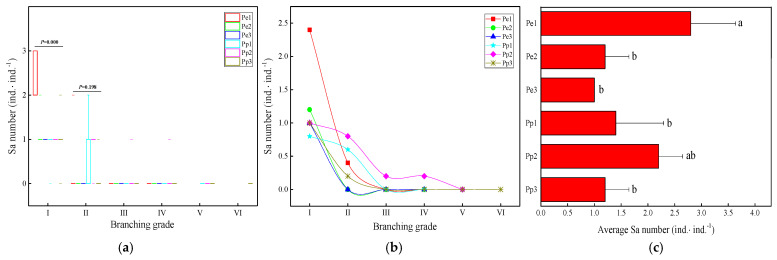
Stand branches (Sa) of ramet systems. (**a**) Number of Sa branches at different branching grades; (**b**) Variation trend of Sa branch number across branching grades; (**c**) Average number of Sa branches in different ramet systems. Different letters indicate significant differences (*p* < 0.05).

Stand branches (Sa) were concentrated exclusively in Grade I and dropped to nearly zero above Grade II (Figure 9a). Pe1 produced substantially more Sa branches than all other treatments (*p* < 0.01). Sa counts declined rapidly with increasing grade for all groups (Figure 9b). Low-density Pe1 and medium-density Pp2 favored Sa formation, while high density drastically suppressed Sa branching (Figure 9c).

**Figure 10:**
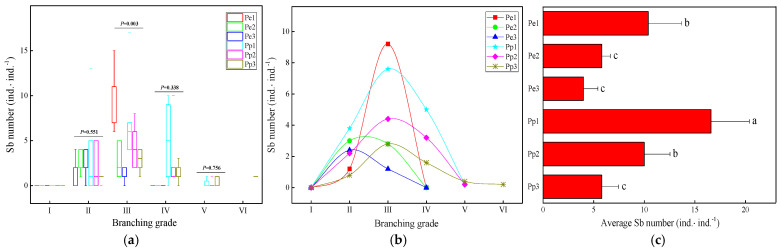
Shoot bud branches (Sb) of ramet systems. (**a**) Number of Sb branches at different branching grades; (**b**) Variation trend of Sb branch number across branching grades; (**c**) Average number of Sb branches in different ramet systems. Different letters indicate significant differences (*p* < 0.05).

Shoot bud branches (Sb) were distributed mainly in Grades II–IV with significant intertreatment differences only at Grade III (*p* < 0.01; Figure 10a). Sb abundance followed a unimodal Grade III peak and vanished after Grade V (Figure 10b). Pe1 formed the tallest Sb peak yet declined rapidly; Pp1 maintained moderate Sb counts with slower decline. Average Sb numbers ranked Pp1 > Pe1 > Pp2, with medium/high densities limiting Sb production in both species (Figure 10c).

### 2.4. Branching Distribution of Mother Bamboo Ramet Systems

**Figure 11:**
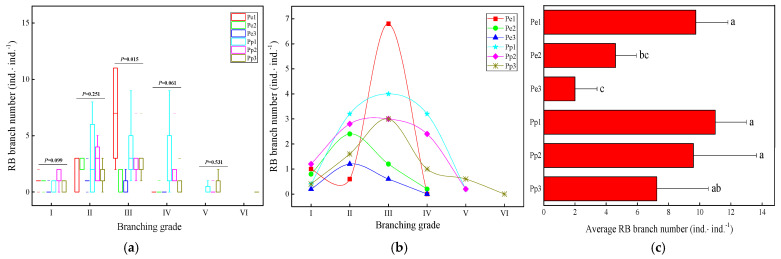
Rhizome basal segment (RB) branching distribution of ramet systems. (**a**) Number of RB branches at different branching grades; (**b**) Variation trend of RB branch distribution across branching grades; (**c**) Average number of RB branches in different ramet systems. Different letters indicate significant differences (*p* < 0.05).

Rhizome basal segment (RB) branches were centered in Grades II–IV and peaked at Grade III (Figure 11a,b). Pe1 had the highest RB branch abundance, followed by Pp1 with a slower post-peak decline. Average RB branch counts were significantly lower in Pe3 relative to Pe1, Pp1, and Pp2 (*p* < 0.05; Figure 11c).

**Figure 12:**
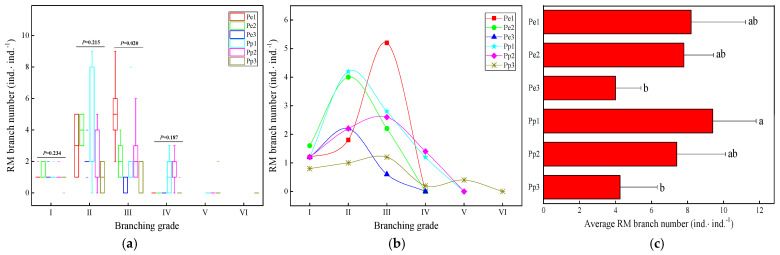
Rhizome middle segment (RM) branching distribution of ramet systems. (**a**) Number of RM branches at different branching grades; (**b**) Variation trend of RM branch distribution across branching grades; (**c**) Average number of RM branches in different ramet systems. Different letters indicate significant differences (*p* < 0.05).

Rhizome middle segment (RM) branching differences were only significant at Grade III (*p* < 0.05), where Pe1 outperformed all other groups (Figure 12a). RM abundance declined sharply after Grade IV (Figure 12b). Low and medium densities (Pe1, Pe2, Pp1, Pp2) generated far more RM branches than high-density treatments, with Pp1 showing the strongest middle-segment branching potential (Figure 12c).

**Figure 13:**
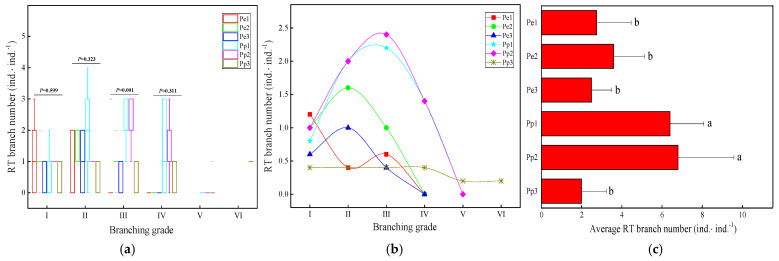
Rhizome terminal segment (RT) branching distribution of ramet systems. (**a**) Number of RT branches at different branching grades; (**b**) Variation trend of RT branch distribution across branching grades; (**c**) Average number of RT branches in different ramet systems. Different letters indicate significant differences (*p* < 0.05).

Rhizome terminal segment (RT) branching exhibited highly significant Grade III differences (*p* < 0.01; Figure 13a). Low/medium Pp (Pp1, Pp2) developed abundant Grades II–IV RT branches, whereas all Pe treatments and high-density Pp3 maintained low RT counts. Pe’s RT peaks occurred at Grade I (Pe1) or Grade II (Pe2, Pe3) with a rapid decline; Pp1 and Pp2 peaked later at Grade III (Figure 13b). Pp1 and Pp2 had markedly higher average RT branches than all other treatments (*p* < 0.05; Figure 13c).

**Figure 14:**
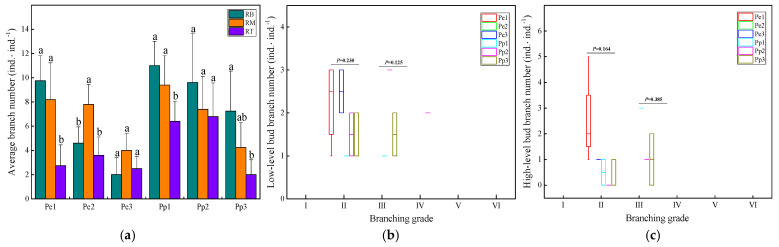
Branching distribution pattern of ramet systems. (**a**) Average branching distribution at different segments; (**b**) Number of low-position bud branches on culm bases; (**c**) Number of high-position bud branches on culm bases. Different letters indicate significant differences (*p* < 0.05). Bamboo culm bases were divided into two equal segments by nodes bearing lateral buds: the lower segment with low-positioned buds and the upper segment with high-positioned buds.

Basal and middle rhizome segments served as primary branching zones, with limited terminal branching (Figure 14a). Low-position culm base bud branches were concentrated in Grades II–IV with no treatment differences (*p* > 0.05), and Pe bore more low-position branches than Pp (Figure 14b). High-position bud branches were rare and restricted to Grades II–III; only Pe1 displayed moderate high-position branching potential (Figure 14c).

## 3. Discussion

### 3.1. Interspecific Differentiation of Underground Architecture in Monopodial Bamboo Ramet Systems

The underground architecture of clonal plants is a core functional trait for adapting to the environment and ensuring population persistence [26] and is strongly regulated by ecological factors such as density and resource availability [27,28]. As a typical gramineous clonal plant, the architectural characteristics of underground rhizome systems in bamboo determine ramet proliferation efficiency and spatial expansion capacity [29,30]. Density, a key population regulatory factor, drives clonal plants to form differentiated strategies between spatial expansion and resource reserves by altering the intensity of intraspecific resource competition, for example, light, water, and nutrient allocation patterns [31,32,33]. In this study, large-diameter *P. edulis* (Pe) and small-diameter *P. praecox* (Pp) were selected to analyze the differentiation of the underground architecture under density regulation via pot-controlled experiments. We found that monopodial bamboos with different culm diameters exhibited significant interspecific differentiation in response to ecological factors such as density, owing to differences in growth requirements and bud bank accumulation traits, forming unique underground architectural strategies. Under high density, Pe’s conservative strategy became more prominent, reducing resource consumption to maintain growth. Meanwhile, Pp strengthened its expansion strategy by sustained branching and rhizome extension to compete for resources, fully verifying the regulatory role of density in the clonal strategies of monopodial bamboos.

This study confirmed that, as a typical large-diameter monopodial bamboo, Pe is characterized by short and thick rhizomes, a low branching hierarchy, and high dormant bud reserves, with rhizome length allocation concentrated in low-to-moderate grades, a significantly larger rhizome diameter than Pp, and much higher dormant bud accumulation under low density. This architectural feature reflects a conservative clonal strategy of resource concentration, conservative expansion, and risk aversion [34,35]. This is concentrated in the development of a few thick rhizomes, reducing unnecessary spatial expansion consumption while reserving abundant dormant buds to cope with environmental fluctuations and competitive pressure, ensuring population stability, and maintaining subsequent growth potential.

In contrast, Pp is a small-diameter monopodial bamboo with slender rhizomes and multi-stage branching. It exhibits high sprouting activity and stable continuous branching. Its rhizome length peak is low and declines slowly, while the dormant bud peak occurs at a later time point. Pp features an extended sprouting period and adopts an aggressive expansion strategy. This strategy includes dispersed resource investment, persistent expansion, and compensatory physiological responses [36,37]. By extending slender rhizomes and increasing the branching hierarchy, Pp rapidly constructs a dense underground rhizome network to occupy space rapidly, while compensating for possible ramet losses through sustained sprouting capacity, adapting to resource competition at different densities.

### 3.2. Competitive Effects of Planting Density on Ramet System Construction

Planting density, a core regulatory factor shaping clonal plant population structure and intraspecific competition patterns [38,39], mediates dynamic differentiation in resource allocation strategies, spatial occupancy patterns, and intraspecific competition intensity throughout the process of ramet germination in early population establishment, clonal system expansion in the mid-stage, and population stabilization in the late stage by altering underground space occupancy, soil nutrient, and light resource allocation patterns [40,41,42]. In this study’s pot density gradient experimental system for Pe and Pp, the two monopodial bamboo clonal plants showed distinct population establishment response trajectories. Low density reduced intraspecific resource competition pressure, facilitating rhizome colonization, dormant bud reserves, and ramet proliferation for the establishment of a bamboo population foundation. Increasing density intensified underground niche overlap, forcing plants to adjust their clonal growth investment ratios, triggering competitive inhibition effects and adaptive compensatory behaviors.

Density is a key factor that regulates ramet system construction in monopodial bamboos. Pe is highly density-sensitive, with optimal growth at low density and significant inhibition of rhizome elongation, nodes, and dormant bud accumulation with increasing density. This reflects its intolerance to crowding and dependence on exclusive resource access. Pp exhibited broad tolerance with optimal growth at medium density and significant inhibition only at high density, with backward-shifted dormant bud peaks and enhanced sprouting compensation at high density. This represents a typical competition–compensation trade-off. Previous studies have confirmed significant ecological niche differentiation and growth differences among clonal plant functional types at different planting densities [43,44]. Pe is a density-sensitive clonal species that prioritizes individual growth over population expansion. Meanwhile, Pp is a density-tolerant species that buffers against high-density growth stress by adjusting the timing of dormant bud germination.

At high densities, both species exhibited significantly reduced branching quantity (Ra/Sa/Sb) and basal/middle branching distribution (RB/RM). This indicated that underground space and nutrient competition preferentially inhibited new branch initiation, followed by rhizome extension and bud bank development. From the perspective of the clonal plant resource allocation trade-off theory [45,46], high population density significantly increases plant maintenance costs. This forces plants to reduce vegetative investment in outward expansion and prioritize resource supply to core surviving organs, such as roots and mature individuals, including altering reproductive strategies [47]. This is consistent with the delayed branching development and inhibited rhizome growth observed in this study.

### 3.3. Hierarchical Regulation of Bud Bank and Branching Pattern in Ramet Systems

The spatial expansion and resource use efficiency of clonal plant ramet systems are regulated by the balance of dormancy and sprouting in underground bud banks and the hierarchical construction of branching patterns. This determines the expansion rate and ecological adaptation strategy of clonal populations via the synergistic coupling of endogenous hormone signals and resource allocation [48,49]. As typical guerrilla-type rhizomatous clonal plants, monopodial bamboos rely more heavily for growth regulation on dynamic turnover of rhizome bud banks and hierarchical differentiation of branches, forming a cascade regulation chain of bud bank–branching–ramet [19]. Our pot density experiment further confirmed that Pe and Pp form clonal strategy differentiation of reserve priority in large-diameter bamboo and expansion priority in small-diameter bamboo via differentiated bud bank activation patterns and branching hierarchy responses. This is consistent with clonal plant risk spreading and resource concentration theories [50,51].

In this study, we found that underground bud banks and branching patterns form the core hub connecting maternal bamboo resource input and ramet system expansion, exhibiting a strict hierarchical distribution: dormant and germinating bud peaks in Grades II–III branches, with branching types (Sa low-position, Sb middle-position, Ra broad-spectrum) and distributions (RB basal, RM middle, RT terminal) differentiated by hierarchy. This forms a construction model of low-position initiation, middle-position expansion, and high-position extension. Gao et al. [25] also found significant differences in rhizome branching quantity and bud density at different grades in P. praecox forests, with low-positioned branches more likely to develop into more ramets, middle-positioned branches responsible for lateral expansion, and high-positioned branches favoring renewal and extension. This is consistent with the hierarchical regulation model in this study. This hierarchical structural regulation optimizes resource transport efficiency within ramet systems and enhances the phenotypic plasticity of clonal populations in heterogeneous habitats.

Pe prioritizes highly dormant buds and low branching continuity, adopting a conservative strategy of reserve priority and delayed expansion. Meanwhile, Pp prioritizes highly germinating buds and multi-stage branching, adopting an aggressive strategy of sprouting priority and continuous expansion. From the perspective of biological growth of monopodial bamboos [21], monopodial bamboos rely on lateral buds on rhizomes to differentiate into new rhizomes and bamboos based on their biological growth characteristics. The activation differences in the culm base lateral buds in this pot-controlled experiment (Pp: mother bamboo + new bamboo; Pe: new bamboo only) further indicated a dormant bud compensatory activation effect during ramet system expansion, with a stronger compensation capacity in small-diameter bamboos. Large-diameter Pe prioritizes dormant bud reserves for growth potential to cope with environmental stress and interspecific competition. This activates a small number of lateral buds only after new bamboo formation, with a conservative expansion rhythm. Small-diameter Pp rapidly mobilizes dormant buds to complete population expansion via the simultaneous activation of multisite lateral buds, with more sensitive and efficient dormant bud compensatory responses.

### 3.4. Limitations and Future Perspectives of This Study

This research adopted a root-restricted pot cultivation experiment. Although this method enables precise control of confounding variables such as planting density, habitat substrate, and water and fertilizer conditions, and allows quantitative observation of bamboo rhizome morphology, underground bud banks, and branching patterns, it is constrained by container space and differs from the habitat of field bamboo forests. However, the previous field surveys conducted by our research team on bamboo ramet systems in field bamboo forests under different management regimes, such as shoot-oriented forests, timber-oriented forests, and intensively mulched forests [19,20], have revealed that field bamboo forests consist of interwoven groups of bamboo ramet systems. Nevertheless, the number of ramet systems is not unlimited; under various management modes, the total number of ramet systems within bamboo forests remains within a relatively stable threshold range. When the number of ramet systems reaches the carrying capacity limit of the site, bamboo forests regulate the quantity of ramet system groups via senescence, mortality, and disintegration of individual ramet systems. This ultimately forms a dynamically balanced growth pattern, where the front segments of ramet systems continuously senesce and die off, while new growth expands at the terminal segments. This indicates that the growth of bamboo ramet systems under natural conditions is also confined by stable spatial, quantitative, and volumetric boundaries, rather than growing without restriction. Against this backdrop, the root-restricted pot experiment adopted in this study can simulate the growth status of ramet systems under the environmental carrying capacity threshold, and effectively explore the growth strategies, density response patterns, and growth limits of ramet systems under spatial constraints and intensified interclonal competition.

In addition, bamboo ramet systems are perennial clonal structures. Their morphological formation, bud bank dynamics, and branching evolution constitute a continuous multi-year dynamic process that undergoes constant adjustments with growth age and environmental conditions. Observation data from only a single growth cycle cannot fully reflect the developmental patterns of ramet systems throughout their entire life cycle. The analysis in this study solely draws on data from the first complete growth cycle, yet the experiment itself was not limited to single-cycle monitoring. To date, our team has carried out continuous tracking surveys of the pot experiment across two full growth cycles. Owing to the massive volume of data, this study prioritizes analyzing the effects of initial planting density on the early establishment stage of mother bamboo ramet systems within the first growth cycle. Beyond the pot experiment, we have simultaneously implemented a long-term fixed-position field trial for newly planted bamboo forests, which has also completed continuous monitoring over two growth cycles. Relying on open wild habitats, this field experiment focuses on investigating the whole developmental and evolutionary process of mother bamboo ramet systems from transplanting to the eventual formation of stable ramet system groups under field conditions.

For follow-up research, our team will conduct long-term fixed-position observations on both pot and field experimental plots, tracking the multi-age dynamics of morphology, bud banks, and branching of ramet systems year by year, to decipher the long-term clonal growth process of bamboo ramet systems. Furthermore, the current research mainly focuses on phenotypic studies, including plant apparent morphology and quantitative traits. In future work, we will carry out multi-dimensional investigations covering rhizosphere microecology, nutrient allocation, and physiological and molecular regulation [52,53], so as to gradually improve the theoretical framework governing the establishment, competition, and coordinated development of ramet system groups in bamboo forests.

## 4. Materials and Methods

### 4.1. Plant Materials

Two typical monopodial bamboo species were selected as the research objects: *Phyllostachys edulis* (Pe), mother bamboo with a breast diameter of 3.5–4.0 cm and a soil ball of approximately 30 cm, and *Phyllostachys praecox* (Pp), mother bamboo with a breast diameter of 2.5–3.0 cm and a soil ball of approximately 20 cm. The bamboo seedlings were introduced from Gaoyun Village, Taihuyuan Town, Lin’an District, Hangzhou City, Zhejiang Province. They were 1-year-old mother bamboos with robust growth, no pests or diseases, and a consistent growth status.

### 4.2. Experimental Design

**Figure 15:**
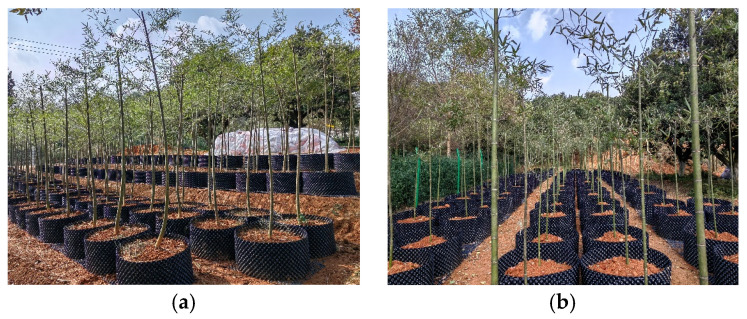
Mother bamboo pots. (**a**) *Phyllostachys edulis* mother bamboo pot; (**b**) *Phyllostachys praecox* mother bamboo pot.

The experiment was conducted in Huizhouzhuang Village, Shuikou Township, Changxing County, Huzhou City, Zhejiang Province, in November 2023. The experimental site has a subtropical monsoon climate, with an average annual precipitation of 1309 mm and an average annual temperature of 15.6 °C. The soil was fertile with sufficient water, which was highly suitable for bamboo growth. A pot experiment with root restriction was conducted (Figure 15), with treatments set for bamboo species and planting density.

Density treatments: Three density gradients were set for *P. edulis*: low density (Pe1), medium density (Pe2), and high density (Pe3), with one, two, and three mother bamboos planted per pot, respectively. For *P. praecox*, the density gradients were low density (Pp1), medium density (Pp2), and high density (Pp3), with the same density settings as *P. edulis*.

Pot specifications and substrate: Root-restricted containers with a diameter of 0.8 m and a depth of 0.5 m were used as pots. Root-restricted material was laid at the bottom of the pots to prevent rhizomes from penetrating the pots into the natural soil. The cultivation substrate was natural soil from a gentle slope at the foot of a bayberry forest, with a uniform texture and consistent fertility.

Experimental arrangement and management: Each treatment had 25 replicates for statistical surveys across the different growth cycles. The mother bamboo plants were planted uniformly in the pots during potting. Natural light was used for all experiments. The pots were thoroughly watered after the initial planting. During periods of water shortage, uniform watering was applied every 3–4 days until water seeped from the pot bottoms and the soil was fully saturated. No additional fertilizer was applied during the experiment to ensure consistent growth conditions across all treatments.

### 4.3. Determination Indicators and Methods

**Figure 16:**
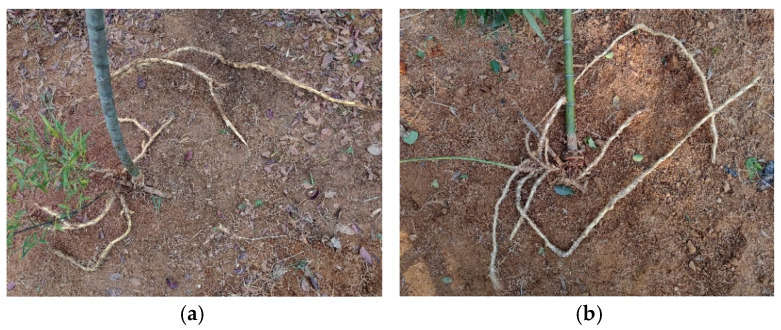
Mother bamboo ramet systems. (**a**) *Phyllostachys edulis* mother bamboo ramet system; (**b**) *Phyllostachys praecox* mother bamboo ramet system.

In December 2024, after the first complete growth cycle of the bamboo plants, five pots, comprising five replicates, of bamboo plants and underground rhizome systems were randomly excavated. The soil and roots were cleaned to maintain the integrity of the ramet system (Figure 16). Rhizomes were classified by branching order. The initial branch of the ramet system was designated as Grade I, and branches sprouting from Grade I branches as Grade II. Rhizome morphology, underground rhizome bud banks, branching type, and distribution were determined.

Rhizome morphology indicators: The length, diameter, and node number of rhizomes of all grades were measured. The percentages of rhizome length and nodes at each grade relative to the total length and nodes were calculated. Average rhizome length, diameter, and number of nodes per ramet system were determined.

Underground rhizome bud bank indicators: The numbers of dormant, germinated, and mortal buds at each branching grade were counted and their proportions at each grade were calculated. The average total numbers of dormant, germinated, and mortal buds per ramet system were calculated. The number of basal lateral buds in the mother and new bamboo was determined.

Branching distribution indicators were the number of rhizome branches (Ra) and rhizome bud branches (Rb), and bamboo stand branches (Sa), Shoot bud branches (Sb) were counted at each branching grade. The Rb branching type was not observed during the survey period. The rhizomes were divided into three equal segments by length: basal segment (RB), middle segment (RM), and terminal segment (RT). The number of branches distributed in the RB, RM, and RT was counted. Bamboo culm bases were divided into two equal segments by nodes bearing lateral buds: the lower segment with low-positioned buds and the upper segment with high-positioned buds. The number of low- and high-positioned bud branches was determined.

### 4.4. Data Statistics and Analysis

Excel 2016 was used for data sorting, and the mean values and standard deviations were calculated. Analysis of variance (ANOVA) and multiple comparisons (SNK test) were performed using SPSS 22.0 software. Graphs were plotted using Origin 2016 software.

## 5. Conclusions

**Figure 17:**
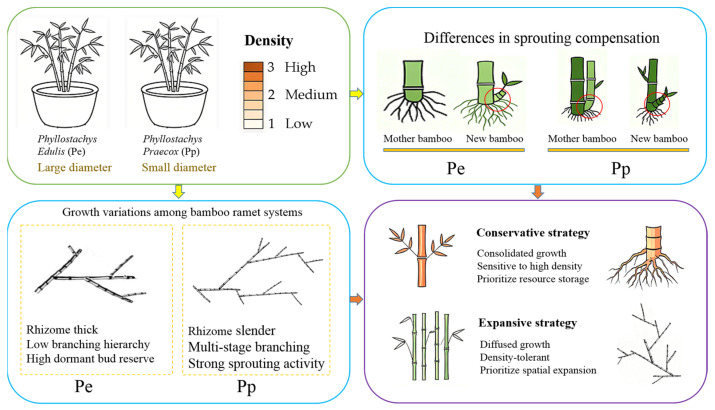
Density-driven ramet system construction in monopodial bamboos shows contrasting responses of *Phyllostachys edulis* and *Phyllostachys praecox* under low, medium, and high initial planting densities. Initial planting density reshapes underground ramet architecture by altering belowground competition. *P. edulis* favors rhizome elongation and dormant-bud accumulation under low density but is strongly inhibited by crowding, whereas *P. praecox* shows stronger sprouting activity and broader branching, with greater tolerance of moderate density.

In this study, *Phyllostachys edulis* (large diameter) and *Phyllostachys praecox* (small diameter) were selected. A pot density control experiment showed construction rules and interspecific differences in rhizome morphology, underground bud banks, branching type, and spatial distribution of mother bamboo ramet systems in typical monopodial bamboos (Figure 17).

*Phyllostachys edulis* adopted a conservative expansion strategy characterized by thick rhizomes, high dormant bud reserves, low hierarchical branching distribution, and density sensitivity, with significantly better performance under low density than under medium and high densities.

*Phyllostachys praecox* adopts a continuous expansion strategy characterized by slender rhizomes, high sprouting activity, high hierarchical branching distribution, optimal growth at medium density, and compensation at high density.

Density inhibits rhizome elongation, branching initiation, and bud bank accumulation in ramet systems by intensifying the intraspecific competition that preferentially affects new branches and high-grade rhizomes. This study was limited by short-term pot control and homogeneous soil conditions. Future research should conduct long-term field positioning observations, ramet system isolation/interaction experiments, and rhizosphere microecology and nutrient allocation studies to further determine the construction, competition, and coexistence mechanisms of bamboo forest ramet system groups. This can provide theoretical support for the sustainable management and precise regulation of bamboo forests.

## Data Availability

Data are contained within the article.
